# Changes in Seroadaptive Practices from before to after Diagnosis of Recent HIV Infection among Men Who Have Sex with Men

**DOI:** 10.1371/journal.pone.0055397

**Published:** 2013-02-06

**Authors:** Snigdha Vallabhaneni, J. Jeff McConnell, Lisa Loeb, Wendy Hartogensis, Fredrick M. Hecht, Robert M. Grant, Christopher D. Pilcher

**Affiliations:** 1 Center for AIDS Prevention Studies, University of California San Francisco, San Francisco, California, United States of America; 2 The Gladstone Institutes, San Francisco, California, United States of America; 3 Department of Medicine, University of California San Francisco, San Francisco, California, United States of America; Asociacion Civil Impacta Salud y Educacion, Peru

## Abstract

**Objective:**

We assessed changes in sexual behavior among men who have sex with men (MSM), before and for several years after HIV diagnosis, accounting for adoption of a variety of seroadaptive practices.

**Methods:**

We collected self-reported sexual behavior data every 3 months from HIV-positive MSM at various stages of HIV infection. To establish population level trends in sexual behavior, we used negative binomial regression to model the relationship between time since diagnosis and several sexual behavior variables: numbers of (a) total partners, (b) potentially discordant partners (PDP; i.e., HIV-negative or unknown-status partners), (c) PDPs with whom unprotected anal intercourse (UAI) occurred, and (d) PDPs with whom unprotected insertive anal intercourse (uIAI) occurred.

**Results:**

A total of 237 HIV-positive MSM contributed 502 interviews. UAI with PDPs occurred with a mean of 4.2 partners in the 3 months before diagnosis. This declined to 0.9 partners/3 months at 12 months after diagnosis, and subsequently rose to 1.7 partners/3 months at 48 months, before falling again to 1.0 partners/3 months at 60 months. The number of PDPs with whom uIAI occurred dropped from 2.4 in the pre-diagnosis period to 0.3 partners/3 months (an 87.5% reduction) by 12 months after enrollment, and continued to decline over time.

**Conclusion:**

Within months after being diagnosed with HIV, MSM adopted seroadaptive practices, especially seropositioning, where the HIV-positive partner was not in the insertive position during UAI, resulting in a sustained decline in the sexual activity associated with the highest risk of HIV transmission.

## Introduction

In the United States, men who have sex with men (MSM) are disproportionately affected by the HIV epidemic. While they represent an estimated two percent of the population, MSM account for over half of all new HIV infections in the United States [1], [2]. “Prevention for Positives” programs aim to include HIV-positive MSM in prevention efforts and help them limit HIV transmission through behavioral and biomedical interventions. The design and implementation of these programs requires a deeper understanding of trends in sexual behavior among HIV-positive MSM over the course of HIV infection.

Previous studies on sexual behavior among MSM after HIV diagnosis found that risky sexual behavior declines soon after HIV diagnosis [3]–[14]. However, a majority of these studies were cross-sectional in nature and were not able to examine trends in these behaviors over time. Studies that do include some longitudinal follow-up suggest a rise in risky sexual behavior 1 year post-HIV diagnosis after the initial drop [5], [10], [14]. Eaton and Kalichman, in their review of sexual behavior across stages of HIV infection, point to the need for longer-term studies on this topic among HIV-infected individuals [15].

It is also important that studies describing trends in sexual behavior among HIV-infected MSM document the increasingly widespread adoption of seroadaptive sexual practices. Community activist groups have defined seroadaptation as modifying sexual behavior based on one’s own HIV serostatus, the perceived HIV serostatus of a sexual partner, and/or differences in risk of transmission by different sexual acts [16]. Seroadaptive practices are based on the idea that new HIV infections do not occur when partners are seroconcordant (serosorting), and that certain sexual positions have higher per-act likelihood of HIV transmission than others (seropositioning or strategic positioning). Unprotected *receptive* anal intercourse has the highest per-contact risk of acquisition when the insertive partner is HIV-positive (at 0.82%), while unprotected *insertive* anal intercourse (uIAI), where the HIV-positive partner is in the receptive role, has a 13.7-fold lower per-contact acquisition risk (0.06%) [17]. In one study, the odds of HIV-positive individuals having uIAI was 13.6 times higher in HIV-positive seroconconcordant partnerships than in partnerships with HIV-negative individuals [18]. Seropositioning among HIV-positive MSM is more common and more successfully adhered to than consistent condom use [19]. Thus, partner serostatus and sexual position are important parameters to consider when characterizing sexual behavior of HIV-positive MSM, especially when one is interested in understanding the prevalence of behaviors that hold high risk for HIV transmission [20].

We undertook the present study to build on this existing literature in two ways: (1) to describe long-term trends in sexual behavior among MSM from before HIV infection to years after HIV diagnosis, encompassing all stages of HIV infection, and (2) to account for the widespread use of seroadaptive practices, especially seropositioning, among MSM when assessing sexual behavior, since different practices carry different risk for HIV transmission. To accomplish this, we modeled the relationship between time since diagnosis and measures of sexual behavior that take into account the number of partners, partner serostatus, and sexual position, among MSM at various stages of HIV infection.

## Methods

### Study Design

Data for this analysis were obtained from The University of California, San Francisco (UCSF) Options Project, a cohort study of individuals who enroll at the time of acute/early HIV infection. Participants are defined as having acute/early HIV if they met one of three criteria: (1) plasma HIV-1 RNA levels ≥3,000 copies/mm^3^ with a negative or indeterminate HIV-1 antibody test; (2) a positive HIV-1 antibody test, with a history of a negative HIV-1 antibody test within the previous 6 months or (3) a clinical history suggestive of recent HIV-1 acquisition, along with a reactive standard HIV-1 antibody test, but a nonreactive less-sensitive (“de-tuned”) HIV-1 antibody test. Once participants are enrolled in the study, they continue to receive routine HIV care through their primary care provider, but also attend study visits at three-month intervals, when blood is collected for a number of tests, including plasma HIV viral load and CD4 count. Details of the Options study eligibility determination and enrollment process are described elsewhere [21]. The Options study protocol was approved by the UCSF Institutional Review Board (Project 10-00301), and written informed consent was obtained from all participants. Beginning in February 2009, behavioral data was obtained longitudinally at three-month intervals starting at the time of the screening visit using Audio-Computer-Assisted Self Interview (ACASI). During the interview, participants were asked about sexual activity in the past three months (or in the three months before diagnosis in the case of the screening visit), including total number of sex partners, perceived serostatus of partners, and types of sexual activity (i.e., insertive versus receptive anal intercourse, and protected versus unprotected anal intercourse). The ACASI typically took 45 minutes to complete. All new participants presenting to the Options study after February 1, 2009 completed an “initial” ACASI at the time of the screening visit, capturing sexual activity in the three months before diagnosis. Subsequently, participants completed “follow-up” interviews at three-month intervals where they reported on those same behavioral variables, with the three-month recall period ending on the day of the interview. Participants who had been enrolled in Options before 2009 completed “follow-up” ACASIs but did not have screening ACASI interviews, since they had enrolled into the study before ACASI was implemented.

It is important to point out here that although the data for this study were obtained from a longitudinal cohort study, the analysis presented below is a series of cross-sectional samples of HIV-positive men at various stages of HIV infection, with follow-up for up to two years for each individual. Each participant interview was indexed by time since diagnosis. A participant who enrolled in Options in February 2009 would contribute data points to the period three months before diagnosis (captured in the initial interview), and at 3, 6, 9, 12, 15 months post-diagnosis (captured through multiple follow-up interviews). A participant who enrolled in Options in February 2005 would contribute data points at 48 months post-diagnosis (since their first ACASI would have been in February 2009, 4 years after their diagnosis), 51 months, 54 months, and so on. The primary analyses focused on population-level trends in the sexual behavior variables described below.

### Study Subjects

Subjects were included in our study if they were enrolled into the larger Options study (i.e., they were 18 years of age or older and met criteria for either acute or recent HIV infection as described above), were male, reported having sex with men in the past three months or self-identified as being gay, and completed at least one ACASI.

### Measures

Participants self-reported their age, race or ethnicity, gender, and sexual orientation. The date of HIV diagnosis was verified in the medical record.

#### Sexual Behavior

At each interview, participants reported the total number of all sexual partners by gender, and the number of HIV-positive, HIV-negative, and HIV unknown-status partners over the three-month recall period. Participants also reported the number of partners of each serostatus with whom they had unprotected insertive anal intercourse (uIAI) and unprotected receptive anal intercourse (uRAI).

#### Clinical Measures

All participants underwent blood testing at the same three-month intervals. Among other tests, CD4 count and plasma HIV viral load (pVL) were obtained at each visit.

### Variables of Interest

Time since HIV diagnosis was the independent variable for this study. The dependent variables we measured focused on sexual behaviors that reflect the number of partners potentially at risk for new HIV infection, either accounting or not accounting for partner serostatus, sexual positioning, and plasma viral load. These include (a) the total number of male sexual partners in the last three months, (b) the number of PDPs (HIV-negative or unknown serostatus) in the last three months, (c) the number of PDPs with whom UAI occurred in the last three months, (d) the number of PDPs with whom uIAI occurred in the last three months, and (e) the number of PDPs with whom uIAI occurred in the last three months while the participant’s pVL was >500 copies/ml.

### Data Analysis

To analyze changes in the number of sexual partners over time, we used negative binomial regression, and modeled the effect of time since diagnosis using a restricted cubic spline. Negative binomial regression was chosen over the Poisson model due to the presence of overdispersion in the data. Zero-inflated negative-binomial models conferred no improvement in fit over regular negative–binomial models. Clustered sandwich estimators were used with the individual as the cluster to allow for within-person correlation in standard error calculations.

A fundamental assumption when modeling our data was that population sexual risk behavior is a smooth function of time. Due to non-linearity in the associations of the independent and dependent variables, a restricted cubic–spline approach was found to best capture the relationship between the time since diagnosis and sexual behavior variables. Lacking *a priori* knowledge of the location of the knots, we constructed five-knot cubic splines by Harrell’s approach of placing knots at the following quantiles of the data: 0.05, 0.275, 0.5, 0.725, and 0.95 [22]. These five-knot spline models were found to be the best fit when compared with spline models with fewer knots as well as with pre-specified knots using Akaike’s Information Criterion (AIC). Graphs were produced from fitted models of transmission risk over time. We present the predicted number of partners and 95% confidence intervals per three-month period for each sexual behavior variable, at specific times during the course of HIV infection.

To assess the impact of outliers on our results, we conducted a sensitivity analysis. We Windsorized the highest values of our partner count data to the 99^th^ percentile, and then secondarily to the 90^th^ percentile, and used these Windsorized variables in our regression models. Graphs and predicted partner counts were compared with corresponding results from our primary analyses.

The relationship between sexual behavior and transmission risk is further influenced by individual infectivity, which is greatly reduced with antiretroviral therapy [23]. To account for the impact of virologic suppression on HIV transmission risk, we assumed that partners of participants with suppressed viral load were not at risk for HIV acquisition. In this analysis, the number of partners at risk for HIV infection was set to 0 if the participant had a plasma viral load <500 copies/ml during the recall period for the interview in question.

### Accounting for Differential Calendar Time at Enrollment into the Study

Study enrollment occurred between 1996 and 2010. During this 14-year period, there were significant advances in the availability of effective treatments and many public health measures directed at changing HIV risk behaviors were put in place. Because of the changing social environment, there may have been differences in baseline sexual behavior between men who enrolled in different calendar years, which could then impact subsequent sexual behavior in the years after diagnosis. For example, those enrolled in 2005 may have engaged in lower levels of risky sexual behaviors to begin with, and may have continued to report lower levels of risky sex in 2009 when they took the ACASI. At the five-year post HIV-infection time point, this would make any noted changes in risk behavior a reflection of the cohort the data are derived from rather than a true decline in sexual behavior in the later stages of HIV infection. To account for this, we compared baseline sexual behavior at the time of enrollment into the study for all participants. We made use of a separate interviewer-administered baseline behavioral questionnaire that was administered to all participants at the time of enrollment between 1996–2008, and the “initial” ACASI interview data for participants enrolled in 2009 or later, to compare mean number of sexual partners and mean number of discordant partners with whom uIAI occurred at baseline. Negative binomial regression models were used to test whether the number of partners reported in the 3-month period before diagnosis changed over calendar time.

All statistical computations were performed using STATA v10.1 (Stata Corporation, College Station, Texas) and SAS v9.2 (SAS Institute, Cary, North Carolina) software.

## Results

A total of 237 HIV-positive MSM were included in the study, and they contributed 504 interviews. Of these, 56 participants enrolled into the Options study after January 1, 2009, and completed a behavioral interview at the time of screening/enrollment into the study. These men contributed data in the pre-diagnosis period and then every three months from that point on, with a median of three interviews (range 1–5) covering a period of approximately 9 months. The remaining 181 men contributed only to “follow-up” interviews at various time points since diagnosis because they were enrolled into the cohort prior to 2009; the median number of interviews in this group was two (range 1–5), covering a period of approximately 6 months. Details of years of enrollment are provided in Table 1.

**Table 1:** Characteristics of HIV-positive men who have sex with men (MSM) in San Francisco in the Options cohort at the time of first ACASI, 2009–2010.

Characteristic		N		(%)
**Gender**	Male	**237**		(100%)
**Age at Diagnosis (Years)**	18–24	**15**		(6.3%)
	25–30	**35**		(14.8%)
	31–40	**119**		(50.2%)
	41–50	**48**		(20.3%)
	>50	**20**	(8.4%)	
**Race/Ethnicity**	White	**165**	(69.9%)	
	Asian	**11**	(4.7%)	
	Black	**15**	(6.4%)	
	Hispanic	**33**	(14.0%)	
	Other	**12**	(5.1%)	
**Year of Enrollment**	1996–2000	**19**	(8.0%)	
	2001–2005	**81**	(34.2%)	
	2006–2008	**81**	(34.2%)	
	2009–2010	**56**	(23.6%)	
**Time Since Diagnosis**	<6 months	**44**	(18.6%)	
	6 months <2 years	**49**	(20.7%)	
	2 years <5 years	**61**	(25.7%)	
	>5 years	**83**	(35.0%)	

Mean age at diagnosis was 37.5 years (SD: 8.9 years). A majority of the participants were White (69.9%), and a majority of the “other” race participants were of mixed Black and Hispanic origin (See Table 1). At first ACASI, nearly one-fifth (18.6%) of the participants had been diagnosed with HIV for less than six months, and one-third (35.0%) had been diagnosed for longer than five years. The median time between estimated date of HIV infection and positive diagnosis was two months (range: 0.5–7 months).

### Trends in Sexual Behavior from before to after Seroconversion

**Figure 1:**
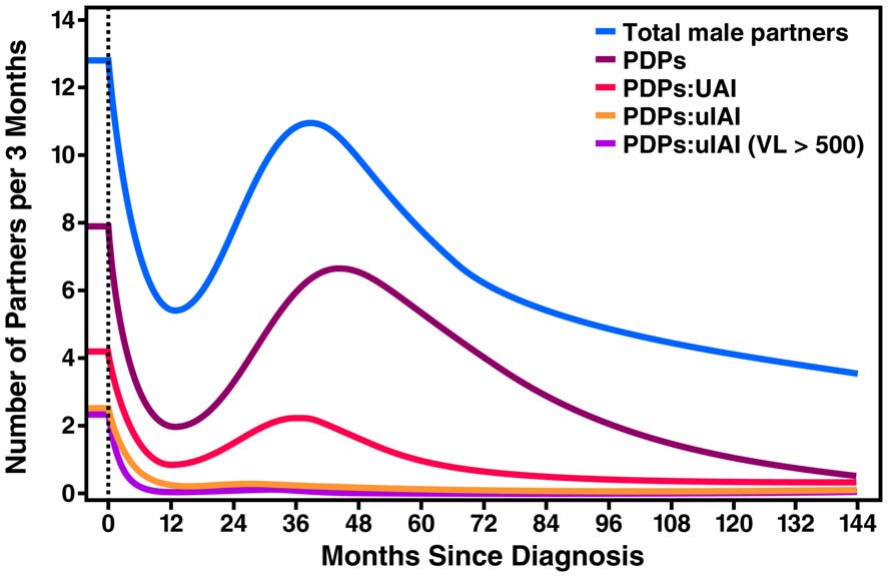
Mean number of partners of various types per 3 months since HIV diagnosis among HIV-positive MSM in San Francisco, 2009–2010. An immediate drop in the total number of male partners in the first year of infection was followed by increases in number of partners over the following 3–4 years. The trend was similar for potentially serodiscordand partners (PDPs) although they comprised only 1/3 to 1/2 of total partnerships. However, unprotected anal intercourse (UAI) with PDPs occurred in far fewer partnerships throughout the follow-up period. Partnerships in which the HIV-positive participant was the insertive partner during unprotected anal intercourse (uIAI) accounted for fewer than 10% of all partnerships and in very few of those partnerships did the participant have sufficient plasma viral load (VL >500 copies/ml) to present a significant transmission risk.

The mean total number of male partners per participant (Figure 1, Table 2) in the three-month period before HIV diagnosis was 12.8 partners (95% Confidence Interval (95%CI): 8.7–18.8). By twelve months after diagnosis, there was a marked decline in the number of partners to 5.5/three months (95%CI: 3.9–7.7), but by 48 months, the total number of partners rose again to 10.0/three months 95%CI: 6.1–16.3). Partner count declined again to a new low of 4.9/three months (95% CI: 3.3–7.4) by 96 months following diagnosis. The mean number of PDPs per three-month period declined from 7.9 (95% CI: 5.7–11.0) in the three months before diagnosis to a low of 2.0/three months (95% CI: 1.3–3.1) at 12 months after diagnosis. We again noted a rise in total number of PDPs to 6.5/three months (95% CI: 3.4–12.7) at the 48-month time point after diagnosis. Similar to the drop in the total number of partners, the total number of PDPs declined steadily after 48 months since diagnosis.

**Table 2:** Estimated mean number of sexual partners per prior 3-month period and 95% confidence intervals [CI] for each sexual behavior variable of interest among MSM in Options/San Francisco, 2009–2010.

Sexual Behavior Variable	Pre-diagnosis N = 54∧	6 months N = 28∧	12 months N = 25∧	24 months N = 74∧	48 months N = 56∧	60 months N = 40∧	96Months N = 25∧
**Total Number of Partners**	**12.8** [8.7–18.8]	**7.1** [5.3–9.4]	**5.5** [3.9–7.7]	**7.8** [5.5–11.1]	**10.0** [6.1–16.3]	**7.8** [4.5–13.4]	**4.9** [3.3–7.4]
**Number of PDP** ∧∧	**7.9** [5.7–11.0]	**3.1** [2.2–4.4]	**2.0** [1.3–3.1]	**3.3** [2.2–5.0]	**6.5** [3.4–12.7]	**5.3** [2.5–11.7]	**2.1** [1.2–3.8]
**Number of PDP with whom UAI** * **Occurred**	**4.2** [2.7–6.6]	**1.5** [0.9–2.4]	**0.9** [0.5–1.7]	**1.5** [0.6–2.5]	**1.7** [0.9–3.1]	**1.0** [0.5–1.9]	**0.4** [0.2–0.8]
**Number of PDP Partners with Whom uIAI** ** **Occurred**	**2.4** [1.3–4.5]	**0.6** [0.3–1.3]	**0.3** [0.1–0.8]	**0.3** [0.1–0.5]	**0.2** [0.1–0.4]	**0.1** [0.1–0.3]	**0.1** [0.04–0.2]
**Number of PDP with Whom uIAI Occurred, Controlling for Viremia** ***	**2.4** [1.3–4.3]	**0.2** [0.1–0.5	**0.1** [0.02–0.2]	**0.1** [0.03–0.3]	**0.04** [0.01–0.2]	**0.01** [0.001–0.1]	**0.01** [0.001–0.03]

∧ Number of participants who contribute to each time point varies because this was not a strictly longitudinal cohort study. All participants started contributing data in 2009, and were at various times since diagnosis when they completed their ACASIs.

∧∧ PDP = potentially discordant partners (HIV-negative or unknown-status partners).

* UAI = unprotected anal intercourse.

** uIAI = unprotected insertive anal intercourse.

*** For participants with plasma viral load <500 copies/ml, number of PDP with whom uIAI occurred was set to 0.

The mean number of PDPs with whom unprotected anal intercourse (UAI) occurred also followed a similar pattern. There was an initial drop from 4.2/three months (95% CI: 2.7–6.6) in the three months before diagnosis to a low of 0.9/three months (95% CI: 0.5–1.7) at 12 months, followed by a rise to 1.7/three months (95% CI: 0.9–3.1) at 48 months, before falling again to 1.0 partners/three months (95% CI: 0.5–1.9) by 60 months.

In contrast to our finding that the above three measures of sexual behavior–namely, total number of partners, PDPs, and PDPs with whom UAI occurred–rebounded after an initial decline in the first year after diagnosis, we found that the mean number of PDPs with whom *unprotected insertive anal sex (uIAI)* occurred declined dramatically, and there was no notable rise over the duration of HIV infection. The number of such partnerships was 2.4 partners/three months (95% CI: 1.3–4.5) in the three months before diagnosis, and this declined sharply to 0.3/three months (95% CI: 0.1–0.8) at 12 months–representing an 87.5% risk reduction in the number of partners at very high risk of HIV acquisition. The number of such partnerships was 0.2/three months (95% CI: 0.1–0.8) at 48 months and 0.1/three months (95% CI: 0.1–0.3) at 60 months.

The mean number of partners at highest risk for HIV acquisition, namely PDPs with whom participants reported uIAI while having a viral load >500 copies/ml, went from 2.4 (95% CI: 1.3–4.3) in the three months before diagnosis to 0.1/three months (95% CI: 0.02–0.2) by 12 months post-diagnosis, and stayed low throughout the duration of HIV infection. This represents a 96% reduction in HIV transmission risk at 12 months when the additive effects of behavioral change and viral suppression were taken into account (assuming that participants with a suppressed viral load do not transmit HIV to negative partners). A sensitivity analysis using Windsorized variables to minimize the impact of outliers yielded comparable results (data not shown).

### Comparison of Baseline Risk Behavior to Assess for Cohort Effect

Pre-diagnosis baseline sexual behavior characteristics, as measured by the mean total number of sexual partners, the mean number of HIV-negative partners, and the mean number of uIAI partners of any serostatus, in the three months before diagnosis, did not differ significantly by year of enrollment into the cohort (reflecting year of infection and diagnosis). The p-values for test-of-trend of mean total number of partners, HIV- negative partners, and mean number of uIAI partners were 0.93, 0.90, and 0.22, respectively.

## Discussion

In this study, we found that MSM in San Francisco with recent HIV infection rapidly changed their sexual behavior after diagnosis in ways that would reduce the likelihood of sexual transmission of HIV to uninfected individuals, and that this effect was sustained over time. Consistent with previous reports, we found that the total number of sexual partners and UAI with potentially discordant partners declined during the first 12 months and then rose at 12–24 months after diagnosis [3]–[14]. However, when the full range of seroadaptative practices, including seropositioning, was taken into account, we found that the behavior that resulted in the highest risk of transmission–uIAI with potentially discordant partners–decreased dramatically in the first year, and continued to stay low over the observation period. These results suggest that HIV-positive MSM may be more likely to adopt seropostioning as a risk reduction strategy rather than serosorting with other positive partners, perhaps because seropositioning allows them to expand their choice of partners while still potentially reducing HIV transmission risk. These findings are consistent with another study among MSM in San Francisco, which found that HIV-positive MSM adopted seropositioning as a risk reduction strategy, in contrast with HIV-negative men, who preferentially adopted a serosorting strategy to reduce their risk of infection [19]. These data suggest a reason for renewed optimism about sustained behavioral change after HIV diagnosis. Even in communities where HIV is endemic and condoms are not used consistently, individuals with HIV infection may be able to reduce transmission risk by adopting and maintaining these seroadaptive practices. These findings highlight the importance of strategies that seek to expand HIV testing and diagnosis, even while working to broaden antiretroviral treatment coverage.

Our study also emphasizes the potential value of *very early* HIV diagnosis–namely, during acute HIV infection–in reducing HIV transmission. The participants in our cohort study were acutely infected and enrolled a median of 2 months after the estimated date of their infection. As shown by Steward and colleagues [24], we found that in the weeks after diagnosis, acutely HIV-infected MSM reduce HIV transmission risk to their partners by adopting seroadaptive practices. Furthermore, this shift in behavior was sustained for many years among our study participants. Since transmission potential is highest during acute infection [25], [26], rapid behavioral change during acute HIV infection has the greatest potential to reduce HIV transmission compared with behavioral change at other stages of infection.

There are many factors that may influence changes in sexual behavior after HIV infection and diagnosis, including age, health status, and access to antiretroviral therapy. Perhaps the most important of these factors may be advancing age. Older age has been associated with a decline in partnership acquisition among MSM (though the decline is less pronounced than among heterosexual men and women) [27]. Although in this descriptive study we were unable to discriminate between the potential reasons for changes in risky sexual behavior, it is important to recognize that participant’s age may have contributed to the decline.

We considered participants with a pVL>500 having uIAI with HIV-negative/unknown-status partners as carrying the highest transmission risk. However, we recognize that transmission of HIV can occur even when the HIV-negative partner is in the insertive position (0.06% per contact risk) [17] and that having an undetectable viral load does not mean HIV transmission will not occur. When used consistently and properly, condoms should always be advocated as one highly effective way to prevent HIV infection and the transmission of some STIs during anal intercourse. In cases where condoms are unacceptable to an individual (or inconsistently used for any reason), transmission may be reduced by advocating for seroadaptive tactics and suppressive antiretroviral treatment among HIV-positive men, with the caveat that successful prevention would be based on disclosure and accurate assessment of partners’ serostatus [28], [29].

This study has several limitations. First, each participant was followed for no more than two years, and our analysis inferred that persons diagnosed with recent HIV infection over more than a decade followed a similar natural history. To address this issue of differences in calendar time at enrollment, we tested for cohort effect to see if those enrolling into the study in different years had different baseline behavior; reassuringly, we found no evidence of a difference. Second, it is also possible that some of the changes in behavior in the cohort might be attributable to differential behavior among those followed and those lost to follow-up and that participants in a study of recently infected individuals may not be representative of the general HIV-infected population. Therefore, we might have overestimated the magnitude of reduction in risky behaviors that occurred over time, relative to baseline. Third, because of a lack of data at certain time points post-HIV infection we had some wide and overlapping confidence intervals, reducing the precision of our estimates of risky sexual behavior, especially many years after HIV infection. Fourth, we measured numbers of partners rather than numbers of sex acts, and so were not able to account for numbers of episodes or acts of uAI with PDPs, and we did not account for whether partners were steady/regular versus casual partners. Finally, the study was not designed to assess the factors motivating seroadaptive behavior (i.e., intentionality, partnership status). Understanding why HIV-infected men do or do not adopt these behaviors–and how these behaviors might be encouraged together with other risk reduction strategies–are important areas for future research. Despite such limitations, results of this observational study provide compelling evidence for behavioral change among the particular cohort of men enrolled in the study (i.e., men diagnosed with acute or recent HIV infection and enrolled in an observational study with frequent and systematic reporting of risk).

Seroadaptive practices, which originated from the community of persons at risk for HIV transmission and not from the scientific or public health communities, reflect tactics that MSM developed as they made choices around sexual partnerships and types of sexual activity. There are serious limitations to the efficacy of these strategies at preventing HIV infection when practiced by HIV-negative men. However, among HIV-positive men, the adoption of seroadaptive practices may be effective at reducing HIV transmission [18], [30]. We observed that HIV-positive MSM in our cohort employed a variety of seroadaptive practices, including seropositioning, to reduce risk of HIV transmission to potentially serodiscordant partners. A further reduction in the number of partners at risk for HIV acquisition was attributable to antiretroviral treatment that effectively suppressed HIV plasma viral load. These findings suggest that improving early diagnosis of HIV infection can empower MSM to further decrease their risk of transmitting HIV, a behavioral change that is achievable. At the same time, our study highlighted the existence of a relatively small pool of men who continue to engage in high-risk behavior after HIV diagnosis. The relevant factors associated with this behavior, and modes to intervene to decrease its prevalence, need to be better characterized. It is likely that reducing HIV transmission rates will require sustained efforts to encourage HIV-positive MSM to engage in regular healthcare monitoring. This will create opportunities for both suppressive antiretroviral therapy and ongoing risk reduction counseling, particularly for those who engage in behavior with high transmission risk. This counseling could include relaying the findings of this paper, as well as estimates of how effective seroadaptation may be for reducing HIV transmission, which might serve to reinforce such behavior among adopters while providing community-tested alternatives to men who have not yet adopted seroadaptive practices. Finally, we note that future studies (including cohort studies, epidemiologic studies and modeling exercises) that assess transmission risk among HIV-positive MSM should account for partner HIV serostatus, sexual position, and HIV viral load in the design of both their questionnaires and models.
